# Value of ^18^F-FDG-PET/CT radiomics combined with clinical variables in the differential diagnosis of malignant and benign vertebral compression fractures

**DOI:** 10.1186/s13550-023-01038-6

**Published:** 2023-10-11

**Authors:** Xun Wang, Dandan Zhou, Yu Kong, Nan Cheng, Ming Gao, Guqing Zhang, Junli Ma, Yueqin Chen, Shuang Ge

**Affiliations:** 1 Department of Medical Imaging, Affiliated Hospital of Jining Medical University, Guhuai Road, Jining, Shandong China; 2 Big Data and Artificial Intelligence, Jining Polytechnic, Jinyu Road, Jining, Shandong China; 3 Department of Radiation Oncology, Affiliated Hospital of Jining Medical University, Guhuai Road, Jining, Shandong China

**Keywords:** Vertebral compression fractures, Radiomics, Positron emission tomography/computed tomography

## Abstract

**Background:**

Vertebral compression fractures (VCFs) are common clinical problems that arise from various reasons. The differential diagnosis of benign and malignant VCFs is challenging. This study was designed to develop and validate a radiomics model to predict benign and malignant VCFs with ^18^F-fluorodeoxyglucose-positron emission tomography/computed tomography (^18^F-FDG-PET/CT).

**Results:**

Twenty-six features (9 PET features and 17 CT features) and eight clinical variables (age, SUVmax, SUVpeak, SULmax, SULpeak, osteolytic destruction, fracture line, and appendices/posterior vertebrae involvement) were ultimately selected. The area under the curve (AUCs) of the radiomics and clinical–radiomics models were significantly different from that of the clinical model in both the training group (0.986, 0.987 vs. 0.884, *p* < 0.05) and test group (0.962, 0.948 vs. 0.858, *p* < 0.05), while there was no significant difference between the radiomics model and clinical–radiomics model (*p* > 0.05). The accuracies of the radiomics and clinical–radiomics models were 94.0% and 95.0% in the training group and 93.2% and 93.2% in the test group, respectively. The three models all showed good calibration (Hosmer–Lemeshow test, *p* > 0.05). According to the decision curve analysis (DCA), the radiomics model and clinical–radiomics model exhibited higher overall net benefit than the clinical model.

**Conclusions:**

The PET/CT-based radiomics and clinical–radiomics models showed good performance in distinguishing between malignant and benign VCFs. The radiomics method may be valuable for treatment decision-making.

## Background

Vertebral compression fractures (VCFs) are common clinical problems that can be caused by various reasons [1–4]. According to the nature of the disease, it can be divided into benign and malignant VCFs. Benign VCFs are caused by osteoporosis or trauma, while malignant VCFs are caused by tumors, such as metastatic solid tumors, myeloma, lymphoma, and Langerhans cell histiocytosis (LCH) [1–3, 5]. With increasing age, benign and malignant VCFs can occur simultaneously [1, 6]. Correct identification of benign and malignant VCFs is of great importance in guiding treatment.

Both open biopsy and percutaneous biopsy are invasive with complications of different degrees. Modern radiological imaging techniques, including computed tomography (CT), magnetic resonance imaging (MRI) and bone single photon emission computed tomography/computed tomography (SPECT/CT), may be helpful in the differential diagnosis based on morphology, signal intensity abnormalities, blood flow and metabolic status. MRI has been established as the most relevant imaging technique for the diagnosis of malignant vertebral lesions due to its high sensitivity to bone marrow abnormalities [7, 8]. Some characteristics commonly associated with malignant fractures include the presence of an epidural or paravertebral soft tissue mass, abnormal signal of the pedicle or other posterior elements, diffuse posterior vertebral border convexity and so on [3, 8]. However, when these typical signs are absent, the diagnosis can be challenging. In addition, there are some drawbacks to MRI, including limited availability, absolute and relative contraindications such as pacemakers and claustrophobia.

^18^F-fluorodeoxyglucose-positron emission tomography/computed tomography (^18^F-FDG-PET/CT) can provide information about both morphological changes and metabolic status of the diseased vertebra. In general, tumor-induced fractures accumulate ^18^F-FDG, while benign fractures are not expected to accumulate the same high level of ^18^F-FDG [1, 9, 10]. This feature is helpful for the quantitative analysis of the lesions. In the study by Won-Ik Cho et al., the threshold of the maximum standardized uptake value (SUVmax) was 4.25 to differentiate malignant and benign VCFs, with 85% sensitivity and 71% specificity for malignancy [10]. In addition, some studies have found that the patterns of FDG uptake such as increased activity involving the pedicle and posterior element are also of great value in differentiating the nature of VCFs [10, 11]. In addition, many quantitative metabolic parameters of PET/CT, such as the peak standardized uptake value (SUVpeak), lean body mass correction of SUV (SUL), and metabolic tumor volume (MTV) have been widely used in the diagnosis, treatment and prognosis assessment of various tumors [12–16]. However, few studies have involved using them in the differential diagnosis of benign or malignant bone lesions [17, 18].

To date, the diagnosis of the causes of VCFs by CT, MRI, bone SPECT/CT and PET/CT is still largely dependent on the experience of diagnostic physicians. Radiomics can extract a large number of quantitative features from digital medical images in high throughput and provide objective information that is difficult for human eyes to quantify [19–21]. Many studies have included reports of the high diagnostic performance of radiomics in distinguishing benign and malignant VCFs using CT and MRI [3, 22].

The objective of this study was to predict the benign and malignant nature of VCFs based on PET/CT radiomics and clinical indicators.

## Materials and methods

### Study participants

**Fig. 1:**
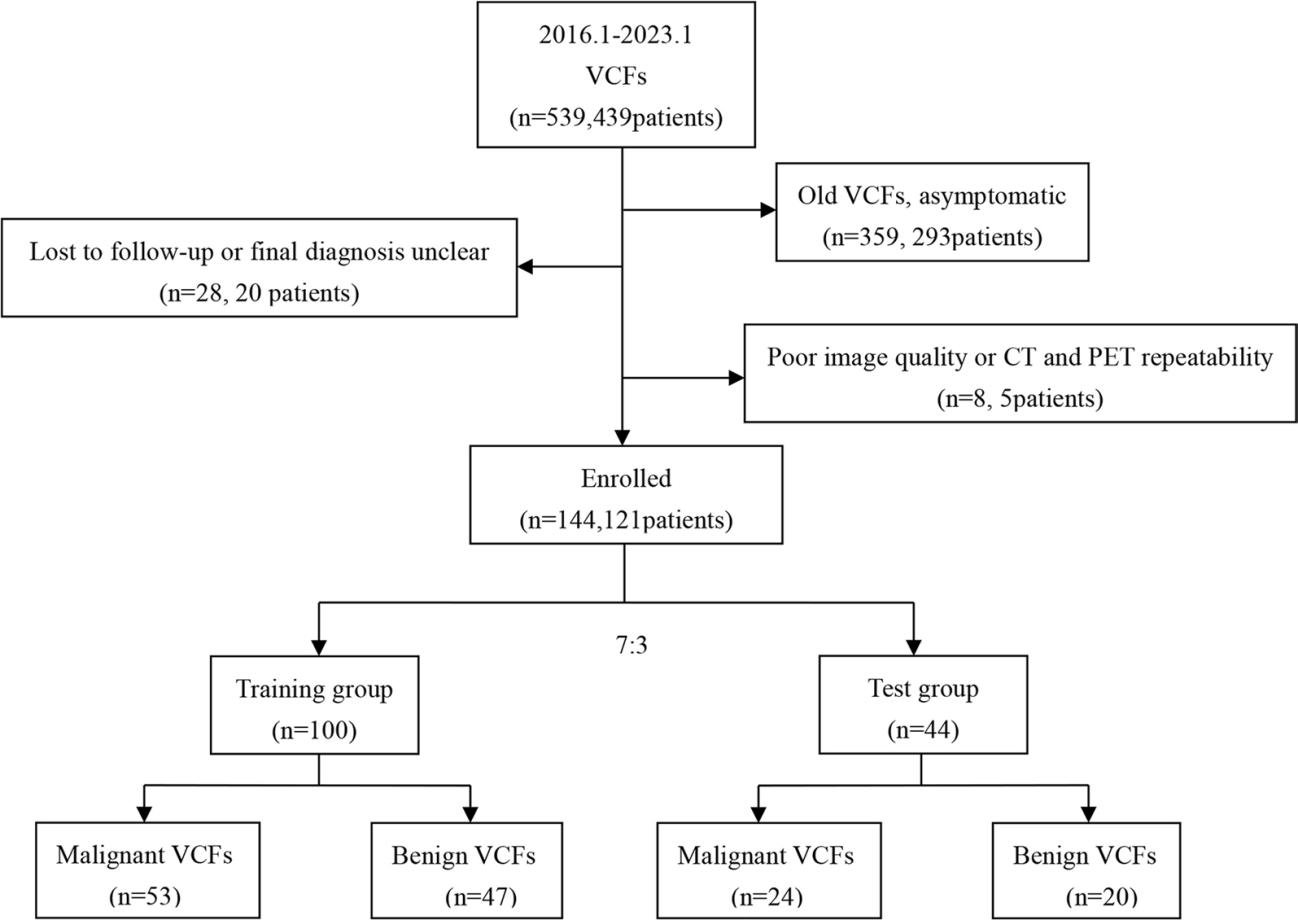
Flowchart of patient screening in this study. *VCFs* vertebral compression fractures

Our institutional Ethics Review Board approved this retrospective study and waived the written informed consent requirement. The analysis was conducted on 439 patients diagnosed with vertebral compression fracture or pathological fracture after PET/CT in our hospital from January 2016 to January 2023, with a total of 539 vertebrae. Vertebral compression fracture was defined as (1) a reduction in vertebral height, (2) cortical discontinuity of an endplate or vertebral cortex with impaction into the vertebral body, or (3) buckling of the vertebral cortex [19, 23]. Patients who had lost follow-up, unclear final diagnosis, asymptomatic old compression fractures, poor image quality or corresponding vertebral body treatment (such as vertebroplasty, kyphoplasty, local radiotherapy of vertebra or systemic chemotherapy) before PET/CT were excluded, and 121 patients with 144 vertebrae were enrolled in the study. All enrolled patients had varying degrees of pain in the corresponding vertebral area within 6 weeks before PET/CT examination. The cohort of patients was randomly divided into a training group (n = 100) and a test group (n = 44) at a ratio of 7:3. There were 47 benign VCFs and 53 malignant VCFs in the training group and 20 benign VCFs and 24 malignant VCFs in the test group (Fig. 1).

### Image acquisition

^18^F-FDG-PET/CT imaging was obtained by a GE Discovery710 instrument, USA. ^18^F-FDG was produced by the GE Minitrace cyclotron and FDG synthesis module, and the radiochemical purity was > 95%. All patients fasted for more than 6 h, and blood glucose was controlled below 11.1 mmol/L before injection. Patients received an intravenous injection of approximately 3.70–5.55 MBq/Kg body weight of ^18^F-FDG, and PET/CT scans were performed from the top of the skull to the upper femur (limbs were scanned if necessary) after a 60 min rest. The CT scanning voltage was 120 keV, tube current was 100 mAs, and the layer thickness was 3.75 mm. PET scanning was performed with three-dimensional acquisition, 2.5 min/ bed, and 5 ~ 7 beds were collected. An iterative method was used to reconstruct the image.

### Clinical data collection, lesion segmentation and radiomics feature extraction

Two nuclear medicine physicians with more than 5 years of experience, who were blinded to the lesion results, analyzed and recorded the lesion features, including lesion site, number, osteolytic destruction, fracture line, soft tissue mass/swelling, and appendices/posterior vertebrae involvement. If there was a disagreement, the decision was made by a third experienced nuclear medicine physician. Multiple metabolic parameters including SUVmax, SUVpeak, SULmax, and SULpeak were measured by creating a region of interest (ROI) along the lesion edge. Data on the patients’ age, sex and history of malignancy were also collected.

**Fig. 2:**
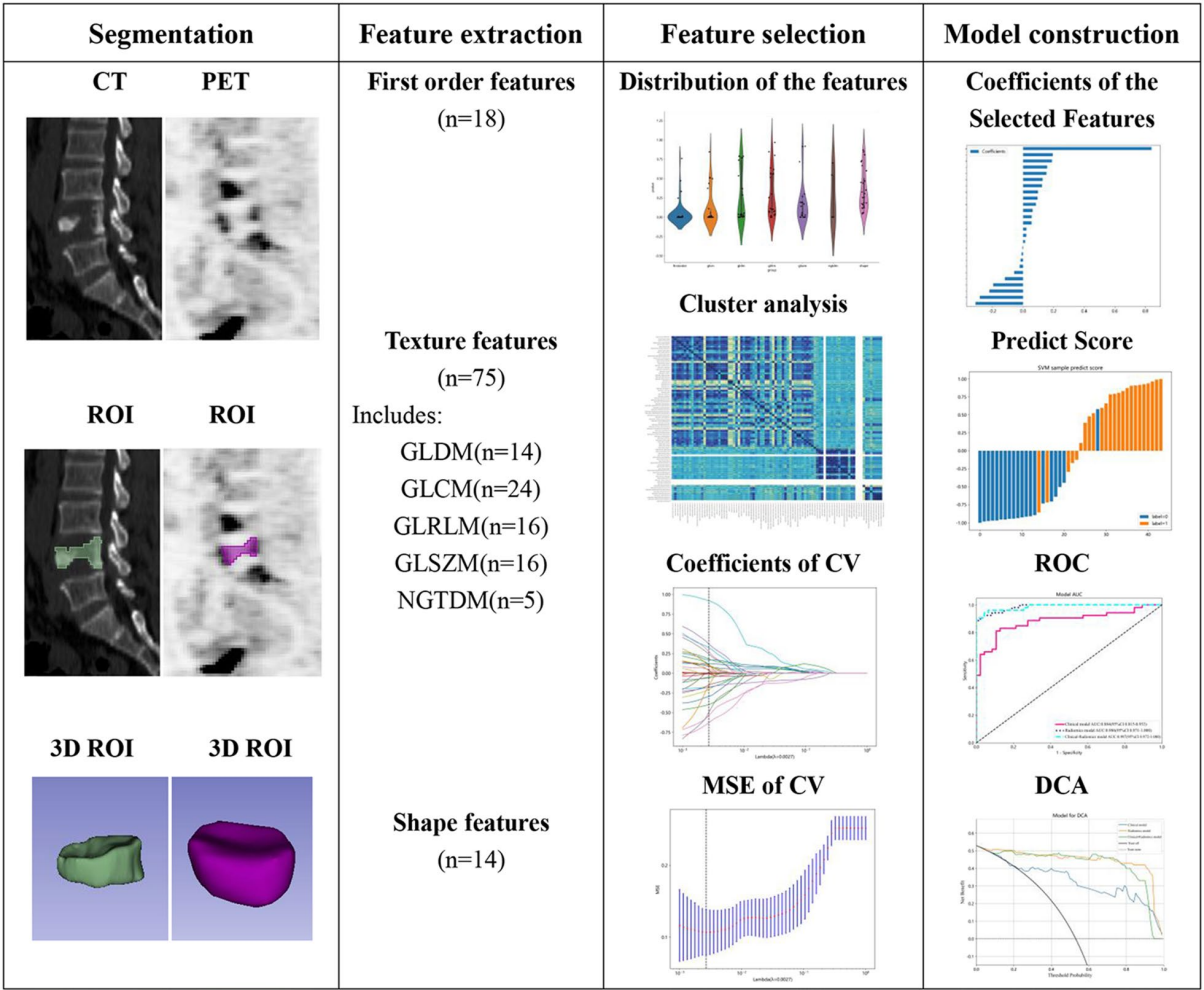
Workflow of the radiomics analysis. *ROI* region of interest. *3D ROI* three-dimensional ROI. *CV* cross-validation. *MSE* mean square error. *ROC* receiver operating characteristic. *DCA* decision curve analysis

The workflow of the radiomics analysis included lesion segmentation, radiomics feature extraction, feature selection, and model construction (Fig. 2). PET/CT data in digital imaging and communications in medicine (DICOM) format were imported into 3D-slicer software (Version 5.1.0, https://www.slicer.org). Two experienced PET/CT diagnostic physicians manually drew the ROI and were blinded to the final diagnosis and clinical history of the patients. For each fractured vertebra, a three-dimensional ROI was drawn along the margin of the cortex of the whole vertebral body and the anterior margin of the bilateral pedicle on sagittal CT images of 3.75-mm thickness. The ROI of PET was sketched along the edges of the ROI of CT. A total of 107 features including 18 first-order features, 75 texture features and 14 shape features were extracted from the ROIs of PET and CT images using 3D Slicer’s built-in module SlicerRadiomics. There are five categories of texture features, including the gray-level dependence matrix (GLDM), gray-level co-occurrence matrix (GLCM), gray-level run length matrix (GLRLM), gray-level size zone matrix (GLSZM), and neighboring gray tone difference matrix (NGTDM). To ensure the stability and reproducibility of the acquired radiomics features, each set of PET and CT image data ROIs was segmented, and radiomics features were extracted twice. The intraclass correlation coefficient (ICC) for each radiomics feature was calculated. ICC > 0.75 was considered indicative of stability, and features were thereby entered into the statistical analysis that followed.

### Feature selection and radiomics model establishment

*Student’s t* test was conducted if the features were consistent with the normal distribution, and the Mann–Whitney*U* test was conducted if the features were not. A value of *p* < 0.05 was considered statistically significant. For features with high repeatability, the Pearson’s standard correlation coefficient (defined as *corr*) was used to calculate the correlation among features. For those pairs of features showing high correlation coefficient (*corr* > 0.9), the feature with a higher *p* value was excluded and the maximum redundancy was deleted each time. Least absolute shrinkage and selection operator (LASSO) regression was applied with penalty parameter tuning by tenfold cross-validation, and features with nonzero coefficients were selected. Finally, the features of CT and PET after screening were entered into a support vector machine (SVM) classifier to establish a radiomics signature that distinguished between benign and malignant VCFs. A radiomics model was built based on both CT and PET findings.

### Clinical model building and clinical–radiomics model

The building process of the clinical signature was almost the same as the radiomics signature. *Student’s t* test was used for normally distribution data, and the Mann–Whitney*U* test was used for nonnormally distributed data. The Chi-square test was used for categorical variables. Statistically significant characteristics were selected. The same machine learning model in the clinical signature building process was used. Then, the clinical signature and radiomics signature were combined to develop a final clinical–radiomics prediction model. A clinical–radiomics nomogram was developed in this study.

### Performance evaluation of the models

The performance of each model was assessed according to the area under the curve (AUC) based on the receiver operating characteristic (ROC) curve analysis, and the sensitivity, specificity, accuracy, positive predictive value (PPV), and negative predictive value(NPV) were calculated in the training and test groups. The Delong test was used to compare the AUC between the three models. Decision curve analysis (DCA) was used to determine the clinical benefit of each model.

### Statistical analysis

Statistical analyses were performed using SPSS (version 20.0; IBM Corp.) and the “Onekey AI” platform (https://www.medai.icu), which is based on PyTorch 1.8.0. Continuous variables are expressed as the mean ± standard deviation(SD), and categorical variables are expressed as absolute counts and percentages(%). Statistical significance was defined as a two-sided *p* value < 0.05.

## Results

### Clinical characteristics

Among the 121 patients, 51 patients were diagnosed with benign VCFs, with 67 vertebral bodies (27 males, 24 females, age 70.6 ± 8.9 years, range 50–92 years), and 70 patients were diagnosed with malignant VCFs, with 77 vertebral bodies (40 males, 30 females, age 60.6 ± 13.4 years, range 13–81 years). The difference between benign and malignant VCFs was statistically significant in age (*p* < 0.05), but not in sex (*p* > 0.05).

All cases were confirmed by histopathology or clinical follow-up examination. Of the 67 benign VCFs, 6 were surgically confirmed and 61 were confirmed by clinical follow-up; among them, 39 cases had a history of malignant tumors. Of the 77 malignant VCFs, 31 were confirmed by puncture or surgery, and 46 were confirmed by comprehensive imaging diagnosis and follow-up. Among them, 64 cases were metastatic solid tumors (28 lung cancers, 7 breast cancers, 6 prostatic cancers, 5 thyroid cancers, 4 colorectal cancers, 4 hepatocellular carcinomas, 2 gastric cancers, 2 renal cancers, 2 esophageal cancers, cervical cancer, ovarian cancer, pancreatic cancer, and synovial sarcoma), 6 cases were multiple myeloma, 5 cases were lymphoma, and 2 cases were Langerhans cell histiocytosis.

### Radiomics feature selection, establishment and performance of the radiomics model

**Fig. 3:**
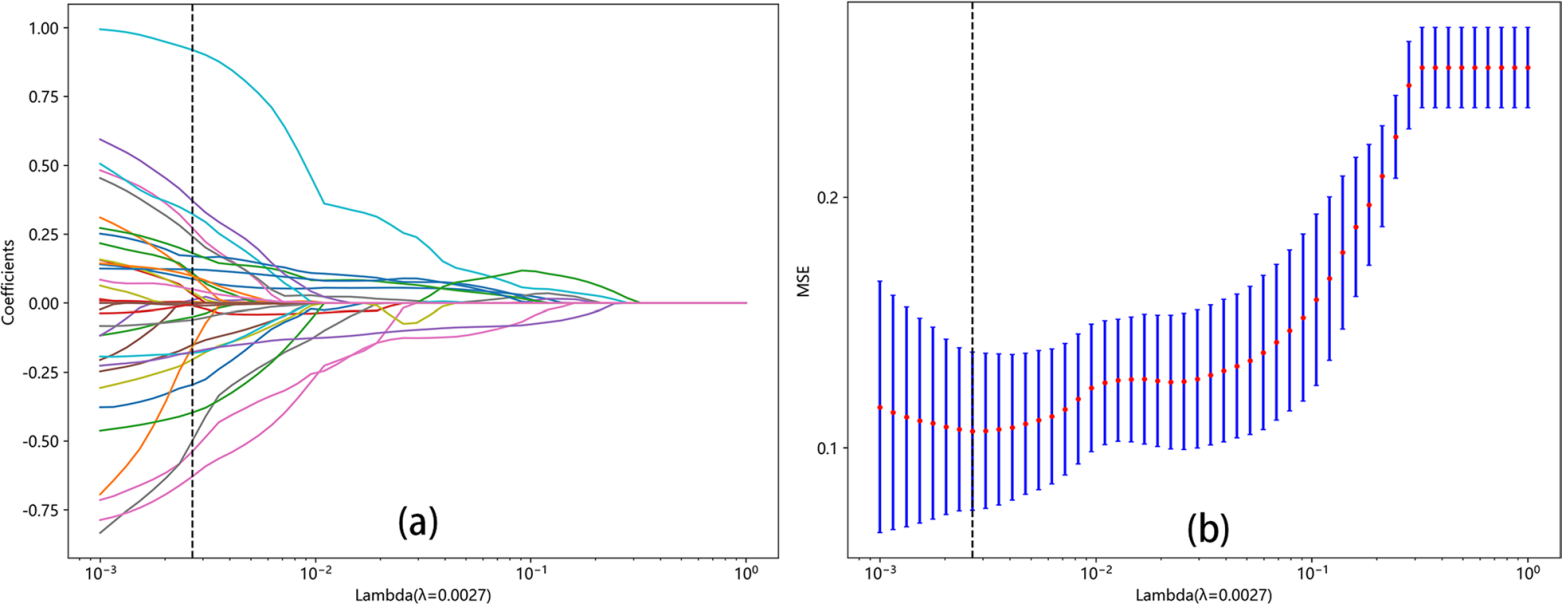
LASSO regression and tenfold cross-validation were used to select the radiomics features. **a** LASSO coefficient profiles of the radiomic features. **b** Optimal feature selection of CV. *LASSO* least absolute shrinkage and selection operator. *CV* cross-validation. *MSE* mean square error

**Fig. 4:**
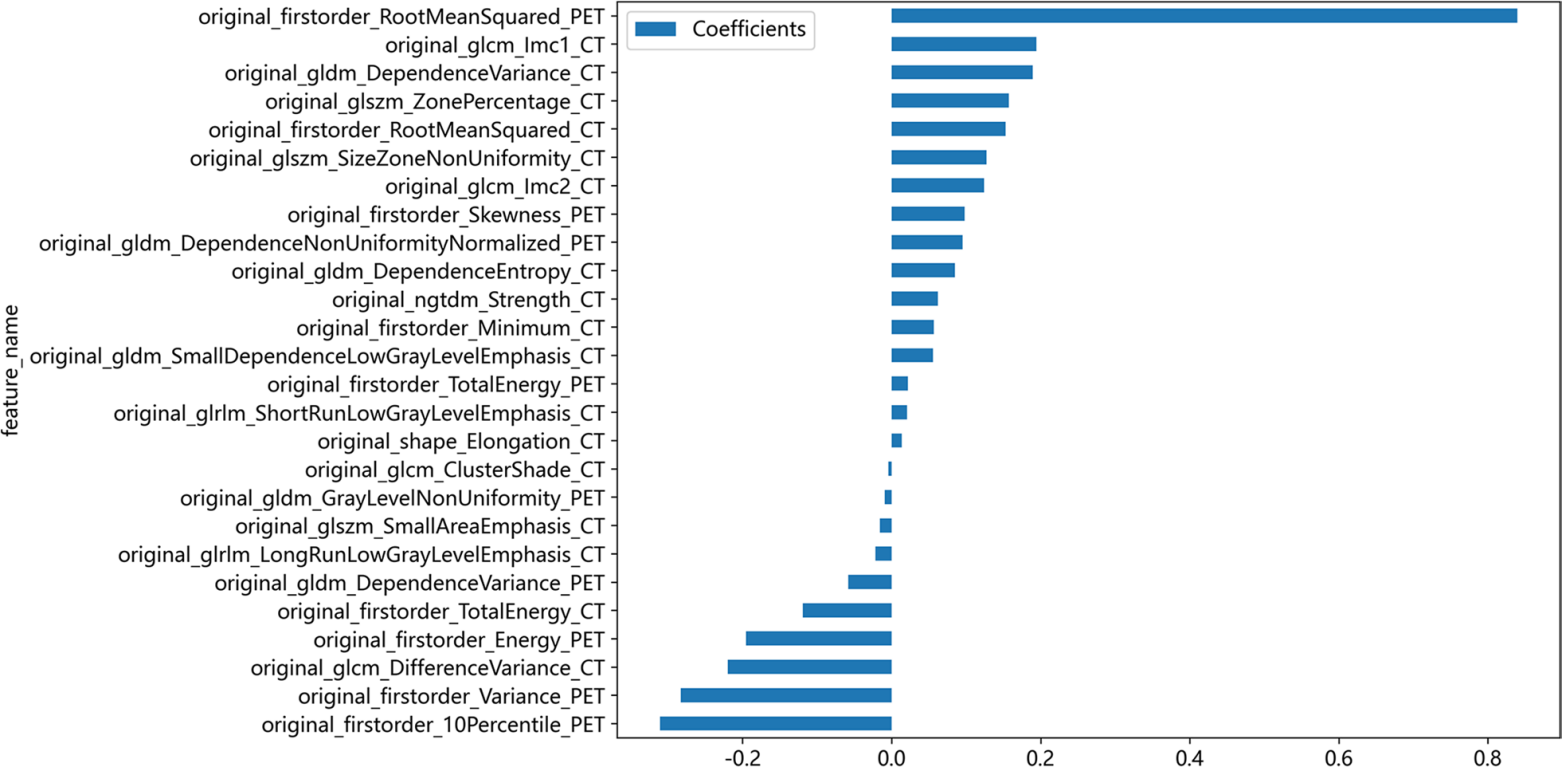
Histogram of the coefficients of the selected features

Twenty-six features, consisting of 9 PET features (6 first-order features and 3 texture features) and 17 CT features (3 first-order features, 13 texture features and 1 shape feature) were selected to construct the radiomics model after LASSO regression and tenfold cross-validation (Fig. 3). The details of the selected features are shown in Fig. 4. The formula of the radiomics signature score (rad-score) for each patient is shown in Table 1.

**Table 1:** Formular of radiomics signature score (rad-score)

Rad-score = 0.5351086867374044	
+ 0.056623*firstorder_Minimum_CT + 0.153041*firstorder_RootMeanSquared_CT − 0.119239*firstorder_TotalEnergy_CT − 0.004347*GLCM_ClusterShade_CT − 0.219531*GLCM_DifferenceVariance_CT + 0.194025*GLCM_Imc1_CT + 0.124174*GLCM_Imc2_CT + 0.085175*GLDM_DependenceEntropy_CT + 0.189177*GLDM_DependenceVariance_CT + 0.055343*GLDM_SmallDependenceLowGrayLevelEmphasis_CT − 0.021720*GLRLM_LongRunLowGrayLevelEmphasis_CT + 0.020565*GLRLM_ShortRunLowGrayLevelEmphasis_CT + 0.127269*GLSZM_SizeZoneNonUniformity_CT − 0.015686*GLSZM_SmallAreaEmphasis_CT + 0.157129*GLSZM_ZonePercentage_CT + 0.062160*NGTDM_Strength_CT + 0.013747*SHAPE_Elongation_CT	− 0.310642*firstorder_10Percentile_PET − 0.195190*firstorder_Energy_PET + 0.839216*firstorder_RootMeanSquared_PET + 0.098178*firstorder_Skewness_PET + 0.021632*firstorder_TotalEnergy_PET − 0.282702*firstorder_Variance_PET + 0.095305*GLDM_DependenceNonUniformityNormalized_PET − 0.057897*GLDM_DependenceVariance_PET − 0.009464*GLDM_GrayLevelNonUniformity_PET

**Fig. 5:**
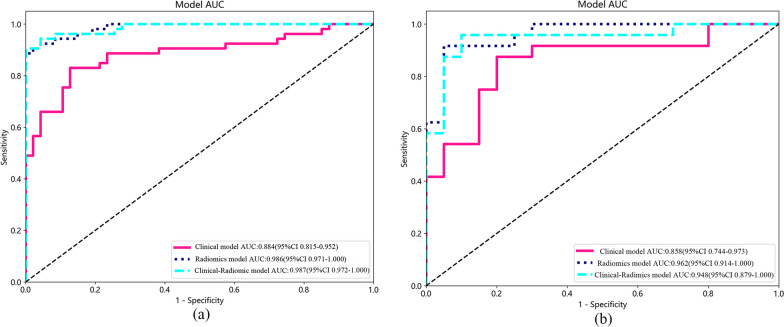
AUCs of the prediction models. **a** The training group. **b** The test group. *AUC* area under the curve

The AUC of the radiomics model for predicting the probability of malignancy of the VCFs was 0.986 (95% confidence interval [CI], 0.9714–1.0000) for the training group and 0.962 (95% CI, 0.9137–1.0000) for the test group (Fig. 5). The accuracy, sensitivity, specificity, PPV, and NPV were 0.940, 0.887, 1.000, 1.000, and 0.887 in the training group and 0.932, 0.917, 0.950, 0.957, and 0.905 in the test group, respectively (Table 2).

**Table 2:** Performance of the prediction models

Prediction model	AUC	95%CI	Accuracy	Sensitivity	Specificity	PPV	NPV	Group
Clinical model	0.884	0.8153–0.9518	0.850	0.830	0.872	0.880	0.820	Train
Radiomics model	0.986	0.9714–1.0000	0.940	0.887	1.000	1.000	0.887	Train
Clinical–radiomics model	0.987	0.9716–1.0000	0.950	0.906	1.000	1.000	0.904	Train
Clinical model	0.858	0.7437–0.9729	0.841	0.875	0.800	0.840	0.842	Test
Radiomics model	0.962	0.9137–1.0000	0.932	0.917	0.950	0.957	0.905	Test
Clinical–radiomics model	0.948	0.8787–1.0000	0.932	0.958	0.900	0.920	0.947	Test

*AUC* area under curve; *CI* confidence interval; *PPV* positive predictive value; *NPV* negative predictive value

### Establishment and performance of the clinical model and clinical–radiomics model

The selection of features for establishing the clinical model was based on a *p* value < 0.05 in the training and test groups. SUVmax, SUVpeak, SULmax, SULpeak, age, osteolytic destruction, fracture line and involvement of the appendices/posterior vertebrae met the conditions and were used to build clinical model (Table 3).

**Table 3:** Clinical variables of benign and malignant VCFs in the training and test groups

Clinical variable	Training (n = 100)			Test (n = 44)		
	Benign (*n* = 47)	Malignant (*n* = 53)	*p* Value*	Benign (*n* = 20)	Malignant (*n* = 24)	*P* Value
SULpeak	2.79 ± 0.90^†^	5.72 ± 2.73	< 0.001	2.73 ± 1.02	5.46 ± 3.08	< 0.001
SULmax	3.45 ± 1.12	7.35 ± 3.59	< 0.001	3.36 ± 1.15	7.16 ± 4.05	< 0.001
SUVpeak	3.58 ± 1.08	7.37 ± 3.50	< 0.001	3.43 ± 1.27	6.92 ± 3.90	< 0.001
SUVmax	4.43 ± 1.35	9.46 ± 4.51	< 0.001	4.23 ± 1.45	9.08 ± 5.08	< 0.001
Age, year	71.68 ± 9.29	59.89 ± 14.40	< 0.001	69.00 ± 8.74	62.25 ± 11.76	0.04
Osteolytic destruction			< 0.001			0.003
No	39 (82.98)^‡^	14 (26.42)		18 (90.00)	10 (41.67)	
Yes	8 (17.02)	39 (73.58)		2 (10.00)	14 (58.33)	
Fracture line			< 0.001			0.025
No	29 (61.70)	51 (96.23)		13 (65.00)	23 (95.83)	
Yes	18 (38.30)	2 (3.77)		7 (35.00)	1 (4.17)	
Soft tissue mass/swelling			0.097			0.974
No	35 (74.47)	30 (56.60)		16 (80.00)	18 (75.00)	
Yes	12 (25.53)	23 (43.40)		4 (20.00)	6 (25.00)	
Appendices/posterior vertebrae involvement			< 0.001			0.034
No	38 (80.85)	15 (28.30)		17 (85.00)	12 (50.00)	
Yes	9 (9.15)	38 (71.70)		3 (15.00)	12 (50.00)	

*SUVmax* maximum standardized uptake value; *SUVpeak* peak of standardized uptake value; *SULmax* maximum lean body mass correction of SUV; *SULpeak* peak of lean body mass correction of SUV

*The differences were assessed by Mann–Whitney *U* test or student *T*-test

^†^Mean ± SD: mean ± standard deviation

^‡^Percentage

The AUC of the clinical model for predicting the probability of malignancy of the VCFs was 0.884 (95% CI, 0.8153–0.9518) for the training group and 0.858(95% CI, 0.7437–0.9729) for the test group (Fig. 5). The accuracy, sensitivity, specificity, PPV, and NPV were 0.850, 0.830, 0.872, 0.880, and 0.820 in the training group and 0.841, 0.875, 0.800, 0.840, and 0.842 in the test group, respectively (Table 2).

The AUC of the clinical–radiomics model for predicting the probability of malignancy of the VCFs was 0.987 (95% CI, 0.9716–1.0000) for the training group and 0.948 (95% CI, 0.8787–1.0000) for the test group (Fig. 5). The accuracy, sensitivity, specificity, PPV, and NPV were 0.950, 0.906, 1.000, 1.000, and 0.904 in the training group and 0.932, 0.958, 0.900, 0.920, and 0.947 in the test group, respectively (Table 2).

### Performance of the prediction models and nomogram construction

**Fig. 6:**
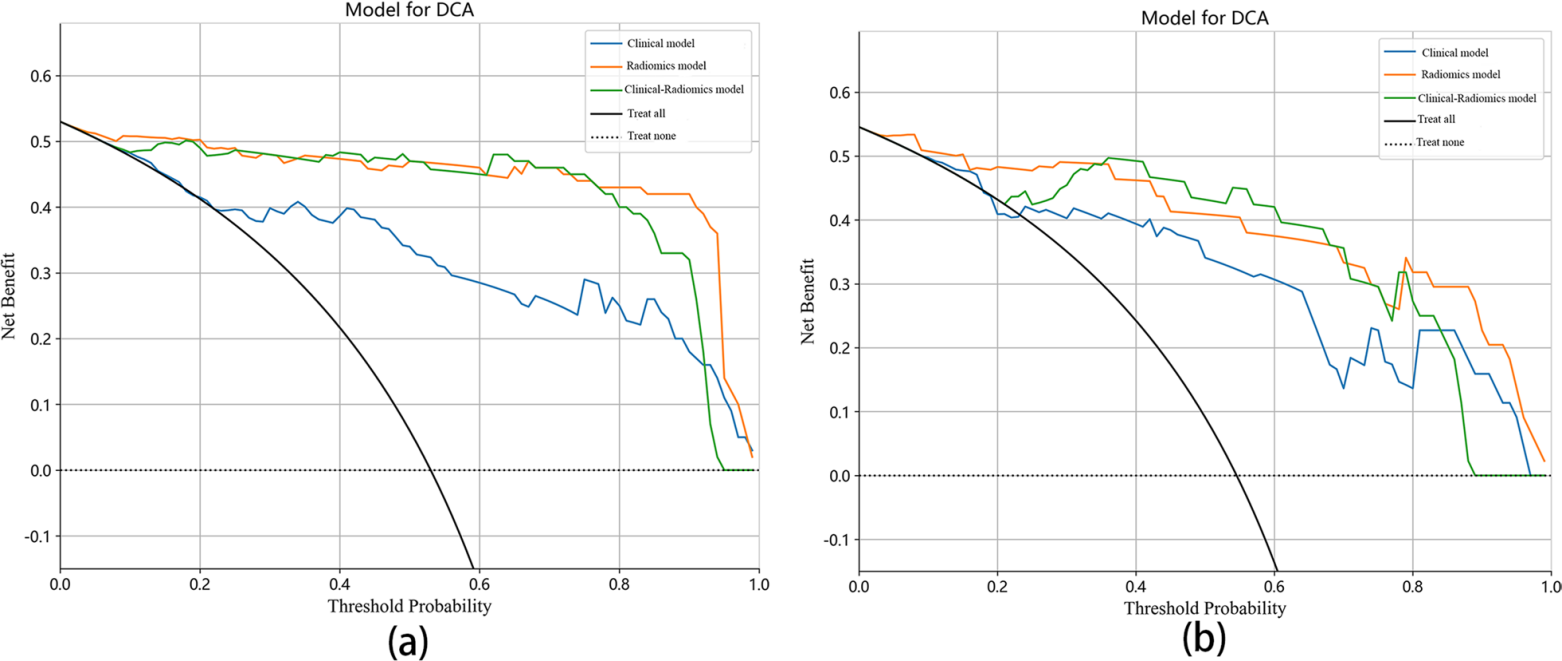
DCA of the prediction model. **a** The training group. **b** The test group. *DCA* decision curve analysis

**Fig. 7:**
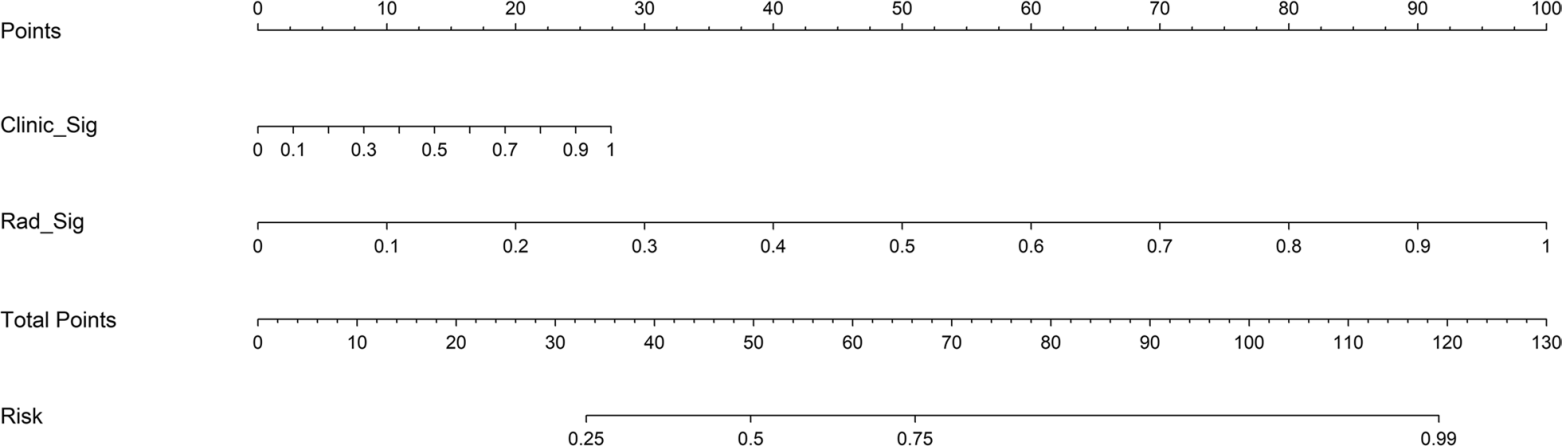
Nomogram to predict the malignancy of VCFs

The clinical model, radiomics model and clinical–radiomics model all showed good calibration. The *p* values of the Hosmer–Lemeshow test for the three models were 0.664, 0.787, and 0.422 in the training group and 0.241, 0.237, and 0.051 in the test group, respectively. The Delong test was used to compare the AUCs of the three models. In both the training and test groups, the radiomics model and clinical–radiomics model were significantly different from the clinical model (*p* < 0.05), but there was no significant difference between the radiomics model and clinical–radiomics model (*p* > 0.05). The DCA demonstrated that the radiomics model and clinical–radiomics model could provide higher overall net benefit than the clinical model (Fig. 6). A nomogram based on the rad-score and clinical risk factors was developed (Fig. 7).

## Discussion

Imaging plays a crucial role in the diagnosis of VCFs. Early and accurate differential diagnosis of benign and malignant VCFs can allow clinicians to effectively choose appropriate treatment plans and potentially provide improved outcomes. Previous studies have reported several CT features that were more frequently found in benign VCFs with statistical significance, including fracture lines, sclerotic bands beneath the end plate, and diffuse thin paraspinal soft tissue thickening [1, 24]. In addition, the intravertebral vacuum phenomenon has never been visualized in malignant VCFs, although its occurrence is uncommon and not statistically significant [1]. CT findings that are predictive of malignant VCFs include osteolytic destruction and epidural or focal paravertebral soft tissue masses [24, 25]. One study showed an accuracy of 89.7% in the differentiation of malignant from osteoporotic vertebral fractures based on the CT scoring system [24]. In our study, osteolytic destruction, fracture line and appendices/posterior vertebrae involvement had statistical significance in differentiating benign and malignant VCFs, but there was no significant difference in soft tissue mass/swelling. A possible reason is that benign VCFs can present as paravertebral or epidural hemorrhage with soft tissue edema. Malignant VCFs usually present as soft tissue masses. However, if there is inflammation around the tumor and/or no tumor infiltrating the cortex, malignant VCFs may also present as similar smooth soft tissue swelling [1]. In our study, we did not distinguish between soft tissue masses and swelling, which might have influenced the results.

Conventional metabolic parameters of PET/CT such as max and peak of SUV and SUL have been used to quantify intratumoral heterogeneity [26]. Some previous studies involved investigating the diagnostic accuracies of PET/CT for differentiating between malignant and benign VCFs and revealed sensitivity ranging from 86 to 100% and specificity ranging from 29.4 to 92.8% [10, 27, 28]. In a meta-analysis, the results reported that PET/CT had excellent diagnostic accuracy in the detection of malignant VCFs, with a sensitivity of 0.96, specificity of 0.77 and AUC of 0.94 [29]. However, a single metabolic parameter (SUVmax) was chosen for analysis in all of these studies. In our study, we chose multiple parameters for analysis. The SUVmax value of benign VCFs was lower than that of malignant VCFs, which was useful in the differential diagnosis of VCFs and consistent with previous studies [1, 29, 30]. The SUVpeak, SULmax, and SULpeak values of malignant VCFs were higher than those of benign VCFs (*p* < 0.05), indicating that malignant VCFs were associated with higher metabolic activity on PET/CT.

Age was significantly different in distinguishing between benign and malignant VCFs. The mean age in the malignant VCF group was less than that in the benign VCF group, which was consistent with previous studies [2, 19, 24]. In our study, the clinical model including age, SUVmax, SUVpeak, SULmax, SULpeak, osteolytic destruction, fracture line and appendices/posterior vertebrae involvement showed a sensitivity of 0.830, specificity of 0.872, and AUC of 0.884 in the training group and sensitivity of 0.875, specificity of 0.800, and AUC of 0.858 in the test group, all of which were values lower than those from the meta-analysis by Kim SJ et al. [29]. This may be because asymptomatic old compression fractures were excluded in our study, and the metabolic parameters of acute VCFs on PET/CT may be higher than those of old VCFs.

Radiomics has been used in many clinical studies, including tumor molecular characteristics, patient prognosis and response to therapy [31]. A number of studies have shown that radiomics and deep learning based on CT and MRI have good diagnostic performance in distinguishing benign and malignant VCFs. The AUCs of the radiomics score on CT for predicting the malignancy probability of VCFs were 0.852–0.97 [19, 25]. The AUC and accuracy of machine learning based on MRI to identify benign versus malignant indistinguishable VCFs were 0.86 and 87.61%, respectively [32]. Liu B et al. believed that automatic deep learning networks showed better diagnostic performance than radiologists in identifying benign or malignant VCFs, and were potentially useful tools for future clinical applications [33]. In our study, we developed and validated a radiomics model and a clinical–radiomics model to predict the malignancy of VCFs on PET/CT. The discrimination performance of the radiomics model and clinical–radiomics model was higher than that of the clinical model in both the training group (AUC: 0.986, 0.987 vs. 0.884, *p* < 0.05) and the test group (AUC: 0.962, 0.948 vs. 0.858, *p* < 0.05).

We believe that radiomics-based models can improve the diagnostic accuracy and efficiency of diagnostic physicians because radiomics models use mathematical algorithms to describe lesions more objectively and can complement radiologists by providing quantitative information that is not available through visual analysis. Radiomics features are believed to reflect intraregional heterogeneity [21, 34]. In our study, the radiomics model included 9 PET features (6 first-order features and 3 texture features) and 17 CT features (3 first-order features, 13 texture features and 1 shape feature). Among the 26 features, the root mean squared, skewness, dependence variance, size zone nonuniformity and dependence entropy were the features with high weight coefficients. Root mean squared as entropy-derived data has emerged as one of the most relevant radiomics features for tumor aggressiveness [35]. The skewness represents the asymmetry of the gray distribution. Higher skewness has been reported as a predictive feature of reduced survival and genetic mutations in lung and colorectal cancer [36–38]. In our study, we found that malignant VCFs had a higher discretized intensity skewness than benign VCFs, which was consistent with the results of Choong Guen Chee’s study [19]. Furthermore, we created a nomogram including the rad-score and clinical risk factors that can depict the prediction results and provide an easy-to-use method for individualized prediction of benign and malignant VCF.

This study has several limitations. First, this was a single-center retrospective study with a relatively small sample size, especially in the test group. Second, most of the malignant VCFs were metastatic tumors, while the proportion of myeloma and lymphoma was small, which might lead to potential confounding factors. Third, the slice thickness of 3.75 mm might be a limitation to this study. Thinner axial slices could increase reliability. Furthermore, we used radiomics for the analysis, and expanding the sample size for deep learning might make the results more reliable and meaningful. Finally, we only performed internal validation. Additional external validation is required to confirm the robustness and generalization of our model.

## Conclusions

In summary, the radiomics model and clinical–radiomics model combining clinical parameters with radiomics scores based on ^18^F-FDG-PET/CT can be used to predict the malignancy of vertebral compression fractures with high diagnostic accuracy. The predictive models can serve as potential decision support tools for clinicians and nuclear medicine physicians and help facilitate the appropriate management of patients with VCFs.

## Data Availability

The datasets used and/or analyzed during the current study are available from the corresponding author upon reasonable request.

## Abbreviations

^18^F-FDG-PET/CT: ^18^F-fluorodeoxyglucose-positron emission tomography/computed tomography
VCFs: Vertebral compression fractures
LCH: Langerhans cell histiocytosis
SUV: Standardized uptake value
SUL: Lean body mass correction of SUV
MTV: Metabolic tumor volume
TLG: Total lesion glycolysis
ROI: Region of interest
DICOM: Digital imaging and communications in medicine
GLDM: Gray-level dependence matrix
GLCM: Gray-level co-occurrence matrix
GLRLM: Gray-level run length matrix
GLSZM: Gray-level size zone matrix
NGTDM: Neighboring gray tone difference matrix
ICC: Intraclass correlation coefficient
LASSO: Least absolute shrinkage and selection operator
SVM: Support vector machine
AUC: Area under the curve
PPV: Positive predictive value
NPV: Negative predictive value
DCA: Decision curve analysis
CI: Confidence interval
OVCF: Osteoporotic vertebral compression fracture
